# Trade-Off vs. Common Factor—Differentiating Resource-Based Explanations From Their Alternative

**DOI:** 10.3389/fpsyg.2022.774938

**Published:** 2022-03-11

**Authors:** Christoph Naefgen, Robert Gaschler

**Affiliations:** Abteilung Allgemeine Psychologie: Lernen, Motivation, Emotion, University of Hagen, Hagen, Germany

**Keywords:** cognitive resources, common factor, trade-off, intra-individual analyses, inter-individual and intra-individual differences

It has long been appreciated that mechanistic explanations of cognition can be tested better when experiment-based studies are complemented with non-experimental studies. For instance, work on executive functions (e.g., Engle et al., 1999; Miyake et al., 2000; Friedman and Miyake, 2004; Oberauer, 2005; Schmiedek et al., 2007) has used between-person variability to more precisely identify candidate mechanisms that explain phenomena related to executive functioning. Notably, such approaches are often focused on analyzing between-person variability rather than on within-person variability. Yet, when seeking mechanistic explanations, cognitive psychologists usually want to know what causes a within-person effect or change (cf. Lewin, 1931; Hommel, 2020a,b), rather than what makes people different from one-another. To counteract risks of ecological fallacy, inquiries should therefore focus on describing and accounting for within-person variability rather than between-person variability. Within-person variability can present as effects of experimental conditions on the individual (rather than group-average effects; cf. Marciano and Yeshurun, 2017) or as spontaneous fluctuation (i.e., day-to-day variability).

Here we present a way of differentiating what we call cognitive resources and common factors from each other using within-person covariance patterns. While several research traditions in cognitive psychology already emphasize within-person variability as a notable phenomenon [e.g., early language development (van Geert and van Dijk, 2002), intermediate phenotypes of ADHD (Castellanos et al., 2005), affect (Eid and Diener, 1999)], we provide some elaboration on uses of within-person variability for readers coming from backgrounds where it usually is not as emphasized (i.e., experiment-based cognitive psychology). Even for those already generally familiar, the specific perspective we describe could be novel, as it focuses co-variation rather than measures of variability. The scope of this paper thus extends to research contexts where theories have a cognitive resource or a common factor as an element and the proposed method serves the purpose of constraining the plausible theory space or to test that aspect of already specified theories (some general areas where this could be useful are attention, executive functioning, dual-tasking).

We link the concept of a cognitive resource to that of a trade-off, signified by negative correlations between two measures on a within-person level of analysis, differentiating it from cases where a common factor is dominant, which results in positive within-person correlations. The two scenarios are described in the following segments and illustrated in Figure 1.

**Figure 1:**
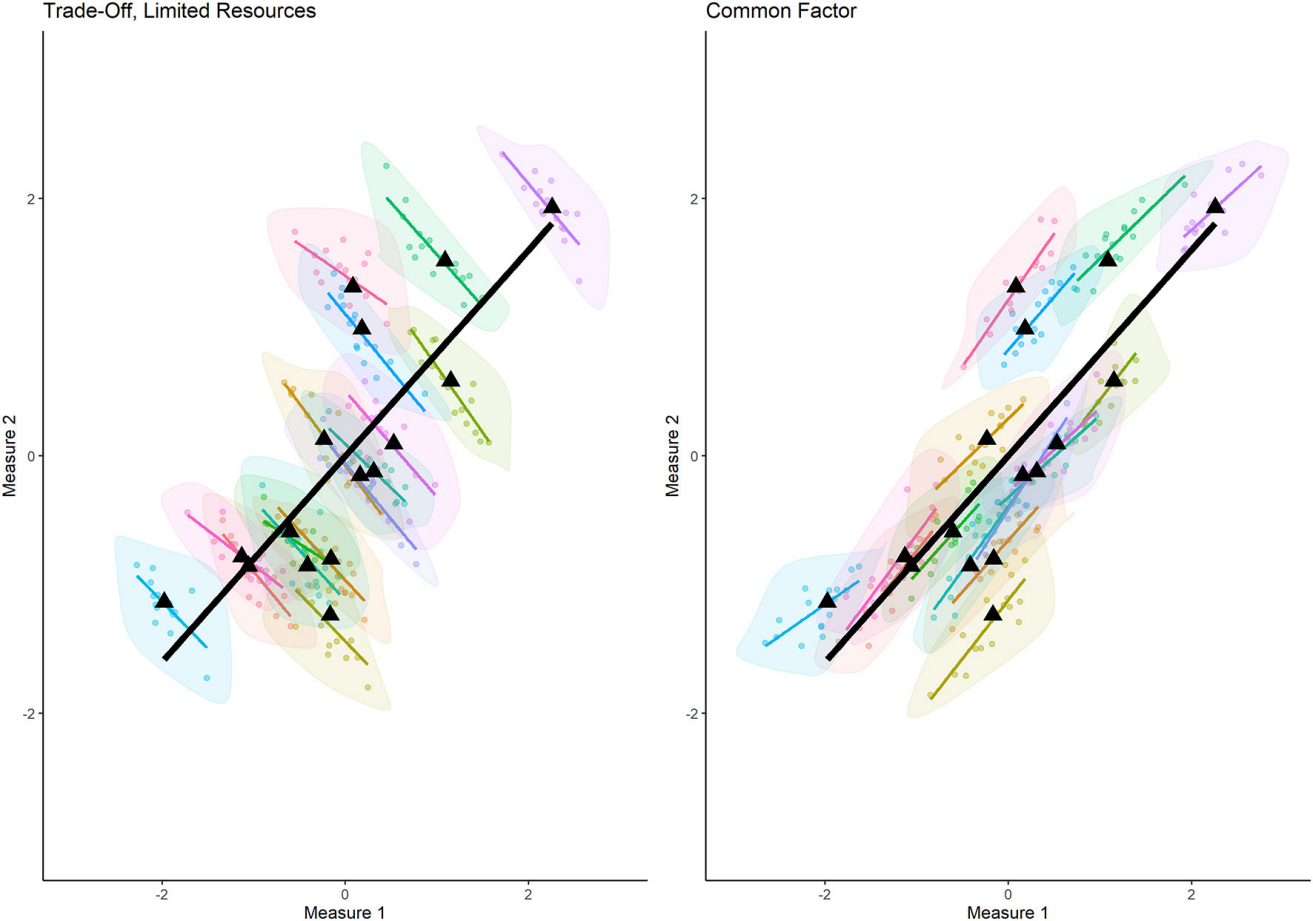
Simulated data illustrating the two scenarios described in this opinion paper that can occur when investigating the correlations between two theoretically-linked measures on a within-person level. The different colors denote different “participants”, the thin colorful lines illustrate the within-person correlations for each simulated participant. The black triangles are places in the mean values for the two measures for each participant and the thick black lines are the between-person correlations resulting out of these averaged values. In the left panel we illustrate what a trade-off scenario would look like and in the right panel what a common factor scenario would look like.

## Short Characterization of the Two Views

### Resource = Trade-Off = Negative Correlation

There are extreme cases where there is little doubt that cognitive processing is characterized by a trade-off. For instance, visual fixations are allocated to one *or* the other of two sufficiently distinct objects in space at a time (e.g., Eriksen and Yeh, 1985; Eriksen and St. James, 1986). Accordingly, allocating the processing of visual information at high acuity to one object necessarily precludes the other object from receiving such privileged processing. Furthermore, the literature on working memory suggests that only one object can be held in the narrow focus of attention in working memory (while approximately four objects can be held in the broad focus of attention) and that shifting the focus from one object held in working memory to another costs time and effort (e.g., Oberauer, 2002; Oberauer and Hein, 2012). This again indicates that granting privileged processing to one object implies withholding it from another object. Maniscalco et al. (2017) reported negative within-subject correlations of performance and metacognition in a vigilance task and used the negatively linked within-session changes to argue for a trade-off and a common resource. Similarly, Drury (1994) wrote about the speed-accuracy trade-off in industrial contexts and the negative correlation between speed and accuracy that appears when people perform resource-limited^1^ tasks.

### Common Factor = Positive Correlation

While a negative within-person correlation suggests the allocation of a limited resource, a positive correlation suggests a common factor. A common factor influences different tasks but is not *limitedly* allocated between the tasks. It can, for instance, be based on (1) a strategy that positively influences different tasks (cf. Gaschler et al., 2014) and is not “used up” when applied to a task), (2) differences in the substrate of the cognitions (e.g., faster overall neuronal transmission rates, Salthouse, 1992), but also (3) environmental factors (e.g., strength of a distraction affecting both tasks). In cognitive psychology, theories explaining cognitive functioning in different tasks by such a common factor are especially prominent in work on cognitive aging, a domain where experimental manipulation cannot be applied broadly and between-person differences as well as trajectories of change within individuals are scrutinized. For instance, based on shared age-related variance across various measures of speed and attenuation of age-correlations after speed-variance is controlled for, Salthouse (1996) has argued that between-person differences and within-person change in the speed of basic processes are common factors behind cognitive aging. Slowing corrupts performance as relevant operations cannot be finished within the available time, products of cognitive processes cannot be combined as results of earlier processing are no longer available when later processes are finished. In a similar vein, based on simulations with neural networks on cognitive aging, Li et al. (cf. Li et al., 2000; Li, 2013; Li and Rieckmann, 2014) have argued for differences in the neural gain parameter as the one common cause of between-person and within-person differences in performance across different tasks.

An example of a common factor is Drury's (1994) observation that providing workers in aircraft inspection with well-defined boundaries to the search area can simultaneously increase speed and accuracy. Workers are thus not just shifted on the speed-accuracy curve in a trade-off manner but their performance benefits overall. As this illustrates, the common factor view is not limited to trait-like factors. This is very important for our argument, because otherwise the contrast we are proposing is not empirically identifiable, as the trade-offs would be visible within-person and the common factors would be visible only between-person. And while this is still possible in cases where a common factor only differs between persons but within each person remains perfectly stable and should be kept in mind, the methods we propose remains viable as long as the common factor has fluctuation in its strength.

On the within-person level, some variables of interest that have been shown to affect performance in tasks commonly used in cognitive psychology are the functioning of working memory, attentional control, motivation (e.g., Adam and deBettencourt, 2019 or Brose et al., 2012), as well as physiological fluctuations such as circadian rhythm, distribution of blood in the body, general stress, or availability of nutrition (e.g., Slaughter, 1901; Hasher et al., 1999). Within-person fluctuations have also been documented for goal-planning (e.g., Wiebe et al., 2018), and self-regulation (e.g., Berg et al., 2014). These variables can lead to positively correlated day-to-day changes in different performance measures.

One thing to mind is that while we use within-person correlations of measurements to illustrate our point, the theoretical conclusions are based on correlations of the underlying *constructs*. Capacity can be distributed to either increase speed or increase accuracy, but the measurement of accuracy can equally be measured in terms of percentage of correct responses or of percentages of errors. The main solutions here, in the spirit of the Research Topic's focus on mechanistic theories and specified cognitive structures, are having a solid theory of how the empirical measurements come about and an awareness that an approach like ours has neither the capacity nor the purpose of replacing good theories. A particularly difficult dynamic here is one in which the distribution of a cognitive resource is governed by a system that can be more or less efficient, that is, a common factor. For example, the action control system can be set to be more flexible or more stable (a cognitive resource, as the control system's balance can only lie in one place at a time) and shifting to flexibility improves task switch performance and worsens repetition performance (and vice versa), but metacontrol adaptivity (how efficient these shifts happen) is a common factor, as it increases the overall performance (cf. Mekern et al., 2019).

A constraint of this approach is that it is tailored to situations in which the amount of available cognitive resources is roughly fixed, at least within the time frame of data acquisition. Once a change in available resources is plausible, the logic we present here is not as easily applied anymore (for one example consider the “less is more” hypothesis of language acquisition, Newport, 1988, 1990, in which an increase in cognitive capacities between early childhood and adulthood leads to reduced language acquisition efficiency since they lead to change in “how” language stimuli are processed).

## Within-Person Covariance as Constraint on Potential Mechanisms

Studies of within-person variability can be used as a first step to constrain the search space for later experimental research to test mechanistic accounts. If it turns out that two measures are correlated negatively within participants (i.e., trade-off), this suggests that processes or representations overlap and are used competitively. An example that most experimental cognitive psychologists will be familiar with is the speed-accuracy trade-off (SAT), which is the phenomenon that for decision making systems the speed with which a decision is made negatively correlates with the accuracy with which the decision is made. Discovered relatively early in the history of modern psychology (Henmon, 1911, for a broader overview see Heitz, 2014), the SAT can, for cognitive psychologists, sometimes be more of a problem to be dealt with (e.g., Vandierendonck, 2018; Liesefeld and Janczyk, 2019), but it illustrates our point about trade-offs well: There seems to be some sort of limit on decision quality gained per time invested, which in accumulator models would be the velocity of evidence accumulation (see, e.g., Bogacz et al., 2010). Shifting toward speed at the cost of accuracy or vice versa results in a negative within-person correlation (Bakdash and Marusich, 2017). As such, the SAT is not only a confound adding noise to be controlled but also an epistemological signal to be used, as it indicates the presence of a cognitive resource.

As an example on the level of task representations, Schuck et al. (2015) used fMRT to track how redundant variants of representing a task as a color- vs. a spatial task were represented and found a negative coupling (space *or* color) rather than redundant coding in task-set relevant brain areas. In the applied domain this might mean that on some days people may approach a traffic light with a task-set strongly weighing color and on other days focusing on light position instead (cf. Overton and Brown, 1957, for a *between*-person difference perspective on this issue). Follow-up work can target active and passive mechanisms clearing redundant parts from task representations. Apart from task sets, trade-offs are also documented on the level of features. A central aspect of the Theory of Event Coding (Hommel et al., 2001) is that features (such as the code “left”) already used for an event file (such as planning a left arm movement) are less available when concurrently needed in a different task (i.e., recognizing a left-pointing arrow, Wühr and Müsseler, 2001).

In case of a positive correlation, candidates for a common factor can be tested. For instance, Brose et al. (2012) documented that days with lower working memory performance, were days with more negative affect and reduced control of attention, suggesting, to follow-up on the role of working memory in emotion regulation.

As mentioned, studies of within-person variability can not only be used to constrain first steps in constructing mechanistic theories, but also to test them. Some theories strongly linked to experimental work can make predictions on the covariance structure of task performance that can be tested in multi-session datasets that allow for day-to-day fluctuation. For instance, bottleneck-theories in dual-tasking (cf. Pashler, 1994; Tombu and Jolicœur, 2005) can be taken to suggest that measures of performance in the two tasks should correlate negatively within subjects.

Lastly, this approach can also be turned on its head to inform how to design environments in which there are multiple related concurrent tasks where good performance in one task is desired and in the other irrelevant. If it is established that a cognitive resource is divided among the tasks, then minimizing the amount of resources used by the irrelevant tasks is a design goal, while if a common factor pattern is established, no such precautions need to be taken. Cognitive load theory in the context of instructional design is an example of this logic: If a student is to learn a subject from teaching materials and needs to use a cognitive resource on both parsing the material and then processing the material, lowering the resource draw of the parsing component will free up resources for the processing component (Sweller et al., 1998).

## Distinguishing Within- and Between-Person Correlation

Correlative studies can help evaluate to what extent mechanisms implying a trade-off structure or mechanisms implying a common factor structure are relevant. Potential outcomes can be that there is evidence only for one or the other case, or that both mechanisms contributed (potentially with different weight). Importantly, to fully harvest the potential of correlative studies for constraining candidate mechanisms, the studies should not be limited to cross-sectional assessment of correlation.

An example involving typing might illustrate that correlations across persons are logically independent from correlations within person (Hamaker, 2012). In cross-sectional studies, the between-person correlation between the time needed for a typing task and error rate might be positive: Some people are good typists. They type quickly and accurately. In contrast, the within-person correlation obtained in a longitudinal study across many typing sessions might be negative: On occasions a person types faster, the error rate will be higher.

A small but growing body of research is now using intensive repeated measures, wherein participants complete tasks on many sessions, to examine within-person coupling of indicators of different cognitive processes. For example, Brose et al. (2012) studied within-person and between-person differences in working memory, control of attention, and affect in 101 young adults across 100 sessions. They obtained evidence for the common factor view (positive correlations), within- and between persons. They found that the same variables that predict between-person differences in working memory performance (cross-sectional correlations) also predict within-person (session-to-session) differences in working memory performance (longitudinal correlations). Given the logical independence of the within- and between-person variability, these results suggest that findings from prior studies of between-person differences that were at risk for ecological fallacy may be at least partially informative about within-person cognitive processes (Molenaar, 2004; Hamaker, 2012).

In summary, we think that the concept of a cognitive resource can be made epistemologically useful and empirically tractable by contrasting it with the concept of a common factor and identifying the two concepts with negative and positive within-person correlations on theoretically related measures. The epistemological use is primarily one of restricting the theoretical space within which mechanistic explanations are to be searched for, but also includes testing hypotheses.

## Author Contributions

CN wrote the first draft of the manuscript and created the figure. All authors wrote and revised sections of the manuscript and read and approved the submitted version.

## Funding

This research was funded within the priority program on multitasking SPP1772 of the German Research Foundation (DFG) awarded to Robert Gaschler (GA 2246/1-2) and supported by the FernUniversität in Hagen.

## Conflict of Interest

The authors declare that the research was conducted in the absence of any commercial or financial relationships that could be construed as a potential conflict of interest.

## Publisher's Note

All claims expressed in this article are solely those of the authors and do not necessarily represent those of their affiliated organizations, or those of the publisher, the editors and the reviewers. Any product that may be evaluated in this article, or claim that may be made by its manufacturer, is not guaranteed or endorsed by the publisher.

## Footnotes

^1^Drury is referring to “task processing resources”, not industrial resources, here.
